# Quantitatively Visualizing Bipartite Datasets

**DOI:** 10.1103/physrevx.13.021002

**Published:** 2023-04-04

**Authors:** Tal Einav, Yuehaw Khoo, Amit Singer

**Affiliations:** 1 Divisions of Computational Biology and Basic Sciences, Fred Hutchinson Cancer Center, Seattle, Washington 98109, USA; 2 Department of Statistics, University of Chicago, Chicago, Illinois 60637, USA; 3 Department of Mathematics and PACM, Princeton University, Princeton, New Jersey 08540, USA

**Keywords:** Biological Physics, Computational Physics

## Abstract

As experiments continue to increase in size and scope, a fundamental challenge of subsequent analyses is to recast the wealth of information into an intuitive and readily interpretable form. Often, each measurement conveys only the relationship between a pair of entries, and it is difficult to integrate these local interactions across a dataset to form a cohesive global picture. The classic localization problem tackles this question, transforming local measurements into a global map that reveals the underlying structure of a system. Here, we examine the more challenging bipartite localization problem, where pairwise distances are available only for bipartite data comprising two classes of entries (such as antibody-virus interactions, drug-cell potency, or user-rating profiles). We modify previous algorithms to solve bipartite localization and examine how each method behaves in the presence of noise, outliers, and partially observed data. As a proof of concept, we apply these algorithms to antibody-virus neutralization measurements to create a basis set of antibody behaviors, formalize how potently inhibiting some viruses necessitates weakly inhibiting other viruses, and quantify how often combinations of antibodies exhibit degenerate behavior.

## I. INTRODUCTION

Given a country’s geographic map, it is straightforward to determine the distance between any pair of cities. Yet, posing this question in reverse (called classic localization or the Euclidean distance geometry problem) is far more challenging: given only the distances between *some* pairs of cities, can we reconstruct the full geographic map [1]?

Across all scientific disciplines, the interactions between vast numbers of entries are routinely measured, yet the deeper relationships underlying these entries only become apparent when recast into a global description of the system [2]. For geographic maps, large tables of city-city distances are less interpretable than a 2D map positioning cities relative to one another.

**FIG. 1.**
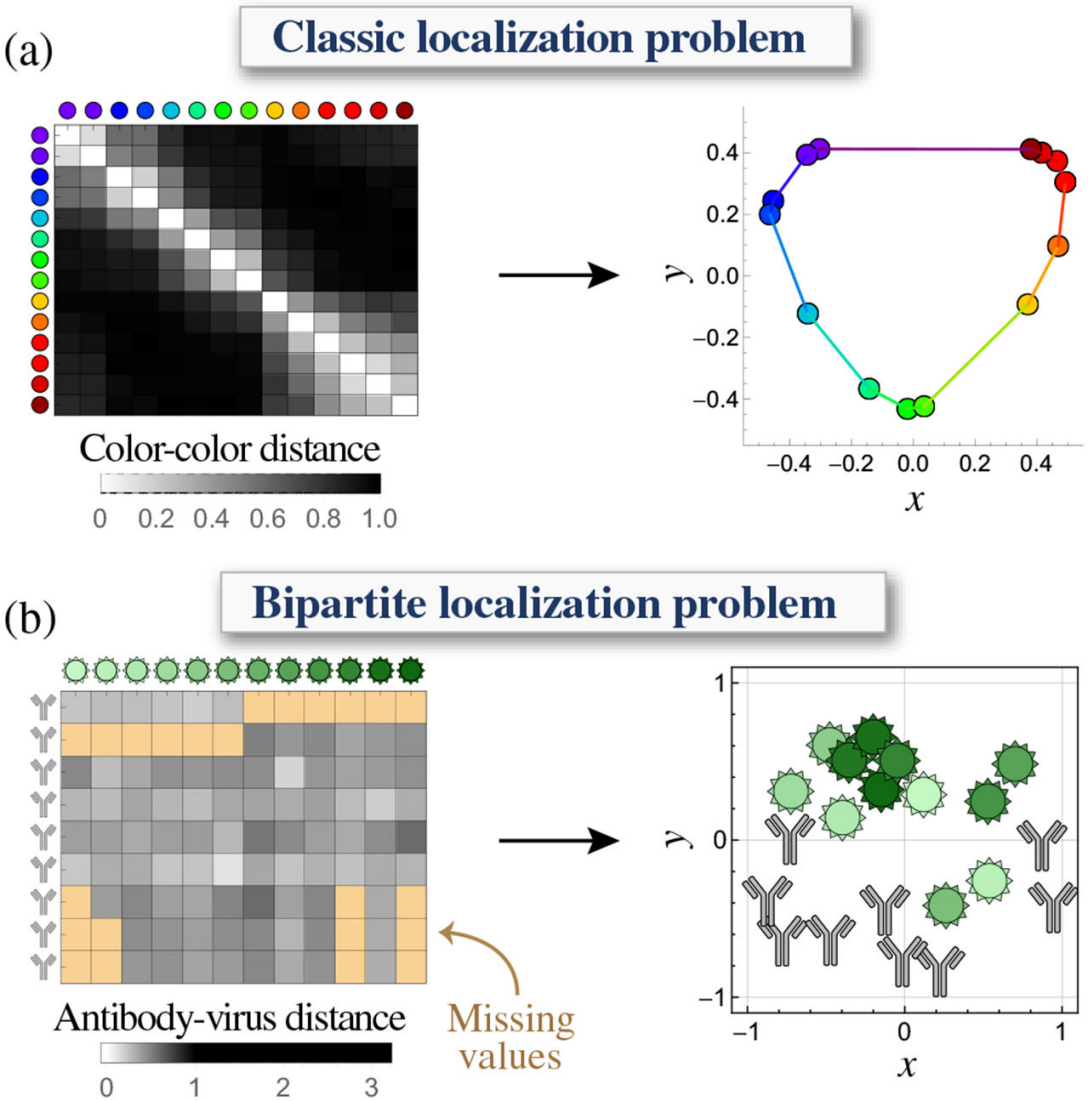
Embedding monopartite or bipartite data in Euclidean space. (a) The perceived similarity between colors recovers the canonical color wheel. Data derived from Table 4.1 of Ref. [3], with distance = 1− (dissimilarities in table). (b) Embedding antibody neutralization against strains of the influenza virus. In this case, only antibody-virus distance can be measured experimentally, and some distances are missing (tan). Viruses are colored from lightest to darkest hues (oldest to more recent strains; full data in Fig. 10 [Appendix A 8]).

To take another example from the field of human perception, the similarity between pairs of colors reveals that reds, greens, blues, and violets cluster together [Fig. 1(a), left]. Yet by embedding these measurements into 2D space (without any additional information about the colors themselves), the colors naturally form into a highly intuitive color wheel [Fig. 1(a), right]. This representation greatly reduces the complexity of the system, enabling us to hypothesize how new colors would be perceived and predict trends in the data (e.g., that each color has a maximally distant “complementary color” on the opposite side of the wheel).

When systems have such a simple underlying structure, we intuitively expect that a straightforward algorithm can dissect the pairwise distances and recover the global embedding. Indeed, for complete and noise-free data this can be achieved in two steps: the first centering the distances to reveal a matrix of inner products, and a second step using the singular value decomposition (SVD) to determine the coordinates (Appendix A 1) [4]. For noisy or partially missing data, numeric minimization [5,6] and semidefinite programming relaxations [7-10] have been developed to drive nonlinear dimensionality reduction [9], nuclear magnetic resonance spectroscopy [11,12], and sensor network localization [6-8,10,13].

In this work, we consider a twist on this classical problem that we call *bipartite localization*, where a bipartite dataset consists of two classes of entries, and interactions can only be measured between (and not within) each class. Since previous methods are poorly suited to handle bipartite data [14,15], we modify existing methods and tailor them for bipartite localization. In particular, we discuss two variants of the popular multidimensional scaling (MDS) algorithm—metric MDS and bipartite MDS—as well as a semidefinite programming (SDP) approach [8]. Each method has its own advantages: metric MDS is the simplest and most flexible numerical framework, bipartite MDS provides a nearly closed-form solution (up to an affine transform), and SDP uses a convex relaxation that is harder to trap in local minima.

Bipartite datasets are ubiquitous in every scientific field, making these embedding methods broadly applicable. Examples include user-rating profiles such as the Netflix challenge [16,17], graph clustering [18-20], the dimensionality of facial expressions [21], the activity of protein mutants [22], gene expression for different DNA promoters [23], and the combinatorics of ligand signaling [24].

As a proof of principle, we apply these methods to the pressing issue of antibody-virus interactions, where multiple antibodies are assessed against panels of virus mutants [Fig. 1(b)]. Unlike many previous efforts that either exclusively visualized the viruses or the antibodies [25,26] or required data to be normalized [27], we embed both types of entries into a shared space that directly corresponds to experimental measurements.

This article explores the underlying computational methods used to create low-dimensional bipartite embeddings, focusing on the effects of noise, missing values, and large outliers. A companion article [28] examines the biological applications in the context of antibody-virus interactions, quantifying the underlying trade-offs and demonstrating how an embedding provides a basis set of antibody behaviors that can dissect the collective response from multiple antibodies. By blending computer science and biophysics, these works show how embeddings collapse the complexity of datasets into a readily interpretable and quantitative framework where key properties such as the potency, breadth, and degeneracy of the antibody response can be rigorously explored.

### A. Need for embedding algorithms

Before exploring the algorithms, we motivate the need for such embeddings by describing several potential applications. To ground this discussion, we suppose the bipartite classes represent antibodies and viruses (with distances describing antibody-virus interactions), although these applications generalize to any bipartite dataset.

First, an embedding combines datasets and predicts unmeasured interactions. For example, we cannot directly compare an antibody measured against viruses 1–6 with a second antibody measured against viruses 7–12 [top two rows in the Fig. 1(b) dataset]. Yet, by embedding both antibodies, we predict their behavior against all viruses in the dataset. Hence, embeddings not only represent a form of matrix completion, but also quantify the similarity between every mapped entity [29,30]. As a point of reference, embedding algorithms assume a different underlying structure for a dataset than low-rank matrix completion, and the combination of the two may be more robust than either algorithm alone (Appendix A 2).

Second, an embedding defines the intraclass distances between any two viruses (or two antibodies), a quantity that by definition cannot be directly measured through antibody-virus interactions. This intraclass distance describes how differently any antibody can neutralize the two viruses (i.e., essentially quantifying their cross-reactivity). In the limit where two viruses lie on the same point, they are neutralized identically by all antibodies; when the two viruses lie far apart, their neutralization can greatly differ.

Third, the inferred virus-virus distances are crucial when designing future experiments. Viruses that are close together offer redundant information, whereas sampling viruses that are spread out across the map can detect more distinct antibody phenotypes.

Fourth, an embedding defines a basis set of behaviors, which is essential for systems where no mechanistic models exist. For example, there is a dearth of models that enumerate the space of antibody behaviors [31-33], which hinders theoretical exploration into features such as the optimality or degeneracy of the antibody response (both of which we address later in this work).

Finally, embeddings provide a fundamentally different vantage to study a system, and this shift in perspective could help uncover its underlying properties. For example, the complex sequence-to-function relationship of viral proteins may be simpler to crack within a low-dimensional embedding. Similarly, quantifying how the antibody response changes with each viral exposure may be more readily understood within the context of an embedding.

## II. ALGORITHMS

We now develop the algorithms to transform pairwise interactions into a global map of a system. In bipartite embedding, we seek to recover the bipartite set of points ${\left\{x_{i}^{*}\right\}}_{i=1}^{m}$, ${\left\{y_{j}^{*}\right\}}_{j=1}^{n}\subset R^{d}$ given the noisy distance matrix $D\in R^{m\times n}$ of the form

(1) $$D_{ij}=D_{ij}^{*}+\epsilon_{ij},\;\;\;\;D_{ij}^{*}=\|x_{i}^{*}-y_{j}^{*}\|,$$

where distance is measured only between the $\left\{x_{i}^{*}\right\}$ and $\left\{y_{j}^{*}\right\}$. $D_{ij}^{*}$ represents the true distance that is perturbed with independently and identically distributed random noise $\epsilon_{ij}$. The goal is to use the noisy $D_{ij}$ with (i,j)∈ℰ, where ℰ represents the subset of measured values, to find an embedding ${\left\{x_{i}\right\}}_{i=1}^{m}$, ${\left\{y_{j}\right\}}_{j=1}^{n}$ that approximates the true embedding $\left\{x_{i}^{*}\right\}$, $\left\{y_{j}^{*}\right\}$. In the following sections, we describe three algorithms to tackle this problem.

### A. Metric multidimensional scaling

Metric MDS consists of the straightforward numerical approach where we randomly initialize each $x_{i}$ and $y_{j}$, and then apply numerical methods (e.g., gradient descent or differential evolution) to match their coordinates as closely as possible to the distance matrix. Through a simple rearrangement of the problem statement, we define the least-squares loss function for metric MDS,

(2) $$\underset{{\left\{x_{i}\right\}}_{i=1}^{m},{\left\{y_{j}\right\}}_{j=1}^{n}}{\text{min}}\sum_{(i,j)\in ℰ}{\left(D_{ij}-\|x_{i}-y_{j}\|\right)}^{2},$$

although we note that other loss functions can strongly affect the embedding (Fig. 11 in Appendix A8).

**Table:** 

Algorithm 1. Classical multidimensional scaling (bipartite MDS).
$$\begin{array}{cc} \; & \;\text{Input}\\ \; & \;\;\;i\;\text{Distance matrix}\;D\in R^{m\times n}\\ \; & \;\;\;\text{ii}\;\text{Dimension}\;d\;\text{of the embedding}\\ \; & \;\text{Steps}\\ \; & \;\;\;1.\;\text{Define a complete distance matrix}\;\tilde{D}\;\text{equal to}\;D\;\text{at measured}\\ \; & \;\;\;\;\;\text{values},\;\text{with missing values filled in using the mean of all}\\ \; & \;\;\;\;\;\text{observed entries in the same row and column}\\ \; & \;\;\;2.\;\text{Compute the double-centered matrix},\;Q=-\frac{1}{2}J_{m}(\tilde{D}\;\circ \;\tilde{D})J_{n}\\ \; & \;\;\;3.\;\text{Compute the top}\;d\;\text{SVD},\;Q={U\Sigma V}^{T}\\ \; & \;\;\;4.\;\text{Set}\;{\left\{x_{i}\right\}}_{i=1}^{m}=U\Sigma A_{U}\;\text{and}\;{\left\{y_{j}\right\}}_{j=1}^{n}=VA_{V}+1{\left(t_{V}\right)}^{T}\;\text{for linear}\\ \; & \;\;\;\;\;\text{transforms}\;A_{U},A_{V}\in R^{d\times d}\;\text{and translation vector}\;t_{V}\in R^{d\times 1}\\ \; & \;\;\;\;\;(\text{where}\;A_{U}A_{V}^{T}=I).\;\text{Determine}\;A_{U},A_{V},\text{and}\;t_{V}\;\text{by minimizing}\\ \; & \;\;\;\;\;\text{the difference between}\;D_{ij}\;\text{and}\;\|x_{i}-y_{j}\|\;\text{using nonconvex}\\ \; & \;\;\;\;\;\text{numerical minimization or SDP (see Appendix}\;A\;5) \end{array}$$

We note that approximate solution methods are necessary because the distance geometry problem is NP hard. To see this, note that embedding a cycle graph in 1D is equivalent to the subset-sum problem, making it NP complete [34]. Bipartite embedding in 1D includes the embedding of an even length cycle, making it NP hard as well.

### B. Bipartite multidimensional scaling

While metric MDS is highly flexible and simple to implement, it does not harness the underlying structure of the bipartite data. In stark contrast, bipartite MDS provides a nearly closed-form solution (up to an affine transform) for noise-free and complete data. Although variants of the classical monopartite problem have been developed to deal with large datasets and noisy measurements [35], to our knowledge this technique has not been extended to complete bipartite data.

The key insight underlying classic MDS is that the *doubly centered* squared-distance matrix is intimately related to the inner products (Gram matrix) of the embedded points. More precisely, we define the centering matrix that subtracts the mean from any vector,

(3) $$J_{k}=I_{k}-\frac{1}{k}1_{k}1_{k}^{T}\in R^{k\times k},$$

where $I_{k}$ is the k×k identity matrix and $1_{k}$ is the all-ones vector of size k (with $J_{k}1_{k}=0$). Consider the complete noise-free bipartite graph,

(4) $${\left(D^{*}\;\circ \;D^{*}\right)}_{ij}={D_{ij}^{*}}^{2}={\left\|x_{i}^{*}\right\|}^{2}+{\left\|y_{j}^{*}\right\|}^{2}-2{\left(x_{i}^{*}\right)}^{T}y_{j}^{*},$$

where ∘ denotes entrywise multiplication. Double centering reveals the inner products of the embedding $X^{*}={\left[x_{1}^{*},\ldots ,x_{m}^{*}\right]}^{T}\in R^{m\times d}$ and $Y^{*}={\left[y_{1}^{*},\ldots ,y_{n}^{*}\right]}^{T}\in R^{n\times d}$ (Appendix A 5),

(5) $$-\frac{1}{2}J_{m}(D^{*}\;\circ \;D^{*})J_{n}=J_{m}X^{*}{\left(Y^{*}\right)}^{T}J_{n}=X^{*}{\left(Y^{*}\right)}^{T}J_{n},$$

where in the second equality we assume without loss of generality that the points in $X^{*}$ are centered at the origin $J_{m}X^{*}=X^{*}$.

The rank-d singular value decomposition of the double-centered squared-distance matrix, ${U\Sigma V}^{T}=-\frac{1}{2}J_{m}(D^{*}\;\circ \;D^{*})J_{n}$, determines the embedding of $X^{*}$ and $Y^{*}$ up to linear transforms,

(6) $$X^{*}=U\Sigma A_{U},$$

(7) $$Y^{*}=VA_{V}+1{\left(t_{V}\right)}^{T},$$

for some matrices $A_{U}$, $A_{V}\in R^{d\times d}$ (satisfying $A_{U}A_{V}^{T}=I_{d}$) and a translation $t_{V}\in R^{d}$ between the centers of $X^{*}$ and $Y^{*}$. Lastly, $A_{V}$ and $t_{V}$ [together with $A_{U}={\left(A_{V}^{T}\right)}^{-1}$] are determined by utilizing the distance information $\|x_{i}^{*}-y_{j}^{*}\|=D_{ij}^{*}$ and minimizing Eq. (2) using semidefinite programming or numeric minimization (Appendix A 5).

In summary, this algorithm reduces the embedding problem with (m+n)d unknown variables into the simpler problem of determining the $d^{2}+d$ unknown variables in $A_{V}$ and $t_{V}$, regardless of the size of D. This same approach can be used for a noisy distance matrix D (Algorithm 1). A caveat of this method is that missing values must be initialized to compute the SVD, effectively adding noise to the distance matrix. Yet the resulting solution may nevertheless approximate the true underlying structure of the system. In the numerical experiments below, we show that although bipartite MDS may yield a poor embedding when a substantial fraction of values are missing, the embedding becomes far more robust when the resulting coordinates are subsequently used to initialize metric MDS (Fig. 12 in Appendix A 8).

### C. Semidefinite programming

Lastly, we investigate an intermediate algorithm that harnesses the bipartite nature of the data to perform a more robust numerical search. More precisely, by forming a positive-semidefinite matrix, we can adapt the sensor network localization SDP algorithm [8] and utilize efficient conic solvers for bipartite embedding [36,37]. We define the combined coordinates $Z=(\begin{array}{c} X\\ Y \end{array})\in R^{(m+n)\times d}$, where X, Y store ${\left\{x_{i}\right\}}_{i=1}^{m},{\left\{y_{j}\right\}}_{j=1}^{n}$. We further define the inner product matrix $G\in R^{\left(m+n)\times (m+n\right)}$ as

**Table:** 

Algorithm 2. Semidefinite programming.
$$\begin{array}{cc} \; & \;\text{Input}\\ \; & \;\;\;i\;\text{Distance matrix}\;D\in R^{m\times n}\\ \; & \;\;\;\text{ii}\;\text{Dimension}\;d\;\text{of the embedding}\\ \; & \;\text{Steps}\\ \; & \;\;\;1.\;\text{Solve}\;G\in R^{\left(m+n)\times (m+n\right)}\;\text{from Eq}.\;(10)\\ \; & \;\;\;2.\;\text{Compute the top}\;d\;\text{SVD},\;G={U\Sigma U}^{T}.\;\text{The embedded}\\ \; & \;\;\;\;\;\;\text{coordinates}\;\{x_{i}\}\;\text{are given by the first}\;m\;\text{rows of}\;{U\Sigma }^{1∕2}\\ \; & \;\;\;\;\;\;\text{while}\;\{y_{j}\}\;\text{are given by the final}\;n\;\text{rows} \end{array}$$


(8) $$ZZ^{T}=\left(\begin{array}{cc} XX^{T} & XY^{T}\\ YX^{T} & YY^{T} \end{array}\right)\equiv \left(\begin{array}{cc} G_{11} & G_{12}\\ G_{12}^{T} & G_{22} \end{array}\right)\equiv G,$$

so that the squared distance between $x_{i}$ and $y_{j}$ can be entirely written in terms of the entries of G, namely,

(9) $${\left\|x_{i}-y_{j}\right\|}_{2}^{2}={\left(G_{11}\right)}_{ii}-2{\left(G_{12}\right)}_{ij}+{\left(G_{22}\right)}_{jj}.$$

Note that we can exactly recast the optimization over X and Y in terms of an optimization over a positive-semidefinite matrix G of rank d. The goal is then to minimize $\sum_{(i,j)\in ℰ}|{\left(G_{11}\right)}_{ii}-2{\left(G_{12}\right)}_{ij}+{\left(G_{22}\right)}_{jj}-D_{ij}^{2}|$ in terms of G. To this end, we introduce an extra error matrix $E\in R^{m\times n}$ and minimize over the sum of errors:

(10) $$\underset{G,E}{\text{minimize}}\;\;\sum_{(i,j)\in ℰ}E_{ij}\;\text{subject to}\;\;E\geq 0,G\;\underline{≻}\;0,\;\;-E_{ij}\leq {\left(G_{11}\right)}_{ii}-2{\left(G_{12}\right)}_{ij}+{\left(G_{22}\right)}_{jj}-D_{ij}^{2}\leq E_{ij},\;\;\;(i,j)\in ℰ,\;\;\sum_{j=1}^{n}G_{ij}=0,\;\;\forall \;1\leq j\leq m.$$

The final constraint ensures that the X coordinates are centered at the origin, removing their translational degree of freedom. Note that to achieve this convex conic program, we remove the nonconvex rank(G)=d constraint, which must now be added back. Thus, we apply a SVD to G of rank d, $G={U\Sigma V}^{T}$. The resulting m+n coordinates are given by $(\begin{array}{c} X\\ Y \end{array})=U\sqrt{\Sigma }$ (Algorithm 2).

As with metric MDS, missing values are seamlessly handled in SDP since the objective in Eq. (10) is restricted to the measured distances. As shown in the following sections, SDP often recovers a better embedding than metric or bipartite MDS, especially when there are many missing values. Note that we specifically choose a different loss function for metric MDS [Eq. (2), optimized for systematic noise] and SDP $\sum_{(i,j)\in ℰ}|{\left\|x_{i}-y_{j}\right\|}^{2}-D_{ij}^{2}|$, optimized to handle outliers) in order to explore the diversity of embedding behaviors. When analyzing datasets, it is worth trying multiple loss functions to determine which one best characterizes the system [Fig. 11(c) in Appendix A 8]. For completeness, we note that bipartite MDS is a nearly closed-form method that does not explicitly use any loss function.

## III. NUMERICAL EXPERIMENTS

We first assess the three embedding algorithms—metric MDS, bipartite MDS, and SDP—using simulated data with m=20 entries $x_{i}$, and n=20 entries $y_{j}$ (each chosen uniformly on [−1,1]×[−1,1]). These points generate the true distance matrix, which we then perturb and use as the input matrix D. The accuracy of the resulting embedding is calculated using the root-mean-square error (RMSE) of Euclidean distances, $\sqrt{\left(\sum_{i=1}^{m}{\left\|x_{i}-x_{i}^{*}\right\|}^{2}+\sum_{j=1}^{n}{\left\|y_{j}-y_{j}^{*}\right\|}^{2}\right)∕(m+n)}$, between the estimated and true coordinates (once aligned via a rigid transform).

### A. Systematic noise and missing values

**FIG. 2.**
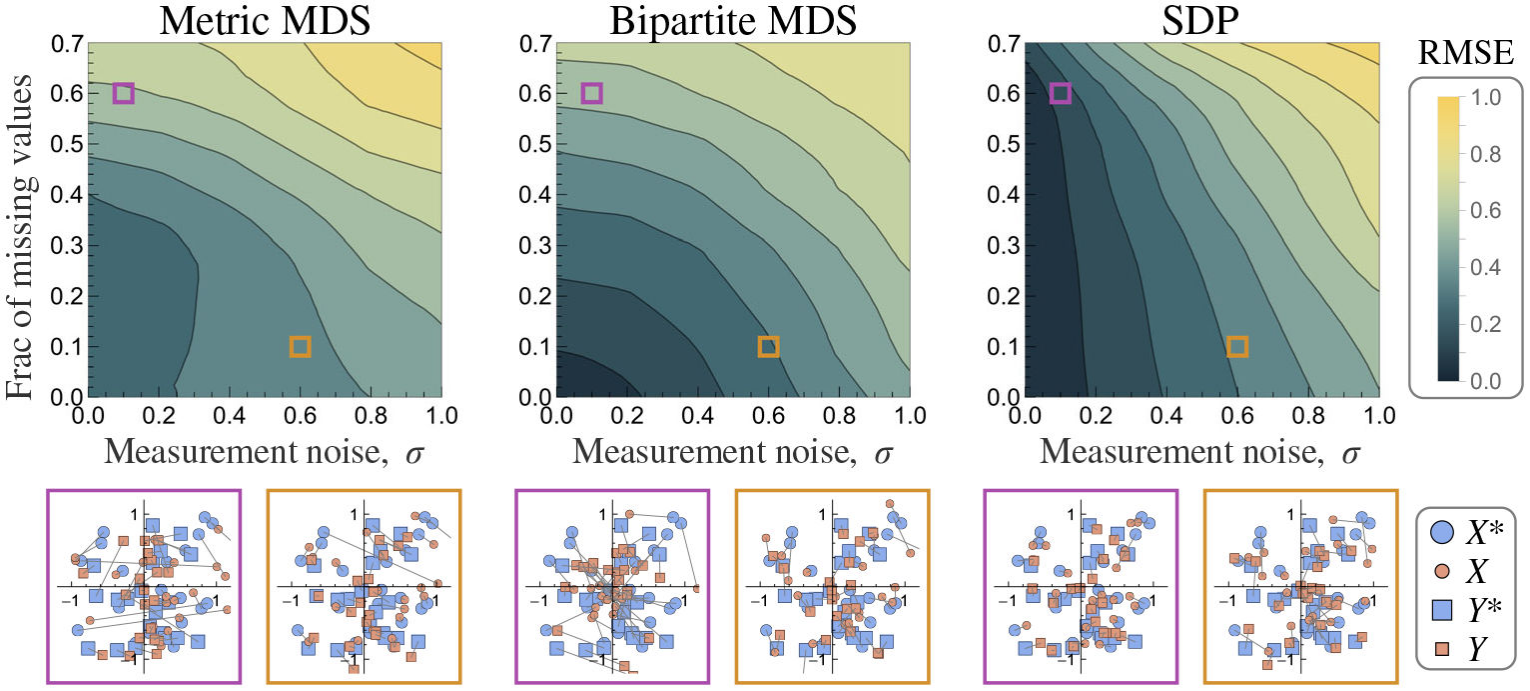
Performance on a simulated dataset. Top: phase diagram of embedding error as a function of the elementwise noise σ of the distance matrix and the fraction $f_{\text{missing}}$ of missing entries for metric multidimensional scaling (metric MDS), bipartite multidimensional scaling, and semidefinite programming (SDP). Error is computed as the average Euclidean distance between the numerical and true coordinates (aligned using a rigid transform). Diagrams show the average of 10 runs, and the metric MDS results were smoothed because its embedding accuracy was erratic. Bottom: examples of the embedding when σ=0.1 and $f_{\text{missing}}=0.6$ (purple box) as well as σ=0.6 and $f_{\text{missing}}=0.1$ (brown box) for each method. Edges connect the numerical coordinates to the true embedding.

To generate the input matrix D, we perturb each entry of the true distance matrix by adding a random value uniformly chosen from [−σ, σ] (x axis) and withhold a fraction $f_{\text{missing}}$ of randomly selected entries (y axis) (see Fig. 2). Of the three algorithms, SDP exhibits the most robust behavior in the presence of missing values, and in the noise-free case along the y axis it undergoes a phase transition from near-perfect recovery when $f_{\text{missing}}\leq 0.6$ to noisy recovery [Fig. 13(a) in Appendix A 8]. In contrast, the error of bipartite MDS increases nearly proportionally to $f_{\text{missing}}$, since each missing value must be initialized as the row or column mean which effectively perturbs the distance matrix. Metric MDS also finds poorer embeddings with larger $f_{\text{missing}}$, as it occasionally gets trapped in local minima (even in the low-noise limit).

When D is fully observed along the x axis, the error increases approximately linearly with noise for all three algorithms [RMSE ≈σ∕2, Fig. 13(b) in Appendix A 8], although metric MDS displays somewhat erratic behavior as it may get stuck in local minima. The bottom panels in Fig. 2 show example embeddings in the intermediate regimes when σ=0.1 and $f_{\text{missing}}=0.6$ (purple) or when σ=0.6 and $f_{\text{missing}}=0.1$ (brown), with gray lines connecting the true coordinates to their numerical approximations.

In terms of overall performance, the region of nearperfect recovery is largest for SDP followed by bipartite MDS and metric MDS (Fig. 2). One way to improve these algorithms is to combine them, for example, by using SDP or bipartite MDS to initialize the coordinates in metric MDS. These combined algorithms substantially improve embedding accuracy, allowing bipartite MDS to handle missing values and extending the capability of SDP to embed noisy measurements (Fig. 12 in Appendix A 8).

Finally, we note that even completely bipartite graphs are not necessarily rigid, and hence multiple incongruent embeddings may describe a dataset equally well. Theoretically, it was shown that when there is no quadric surface separating the two sets of points, then a bipartite graph is universally rigid [14]; in other words, rigidity not only depends on a graph’s connectivity, but also on the resulting positions of the points. As a proxy for rigidity, we can use the rank of the positive-semidefinite matrix G from SDP (Fig. 9 in Appendix A 8).

### B. Handling large outliers and bounded measurements

**FIG. 3.**
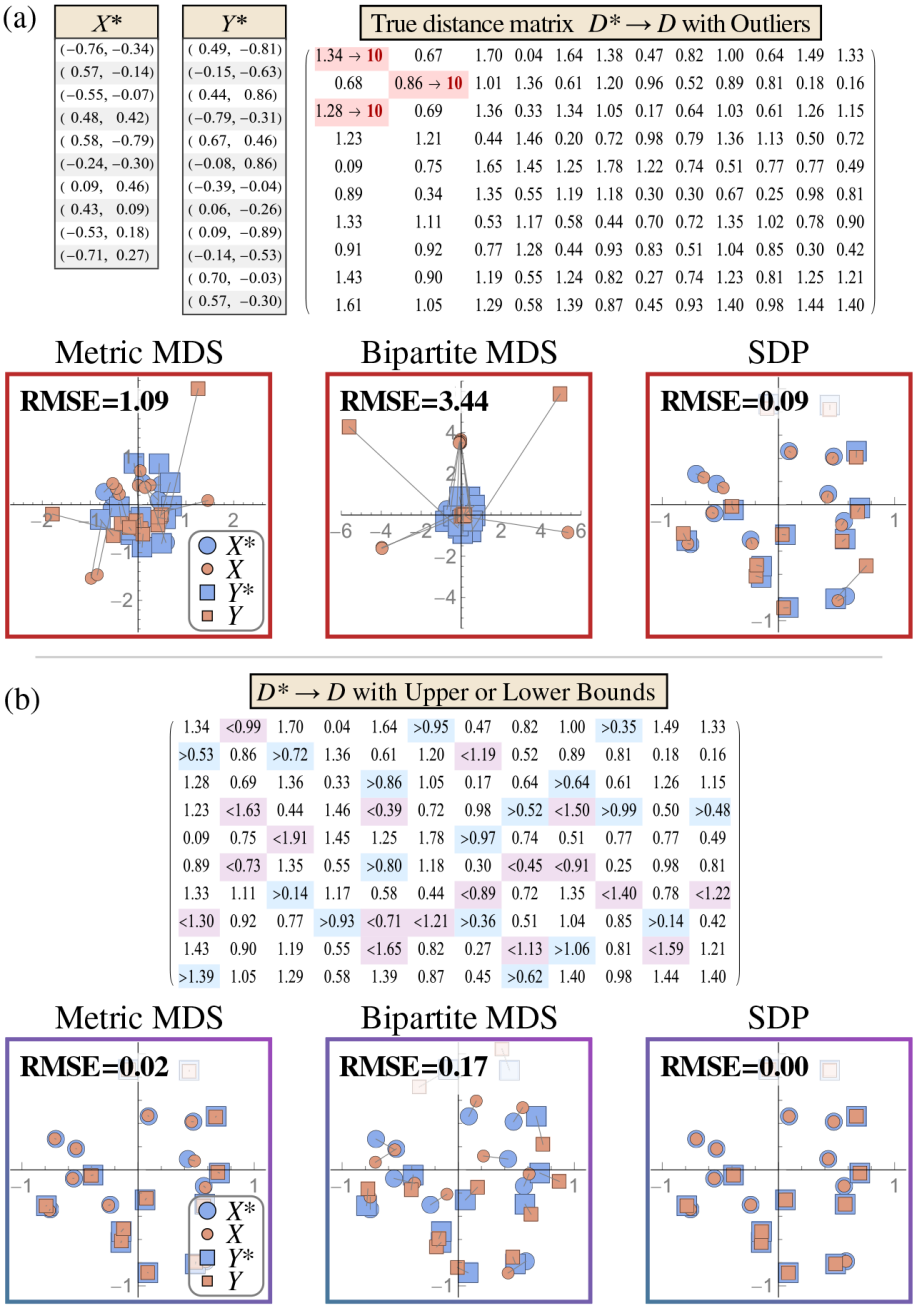
Embedding with outliers and bounded data. (a) Embedding a noise-free distance matrix D with three highly corrupted measurements (highlighted in red). (b) Embedding a distance matrix where 30% of entries are replaced with upper or lower bounds (blue and purple).

In addition to noisy measurements, datasets may contain outliers that distort an embedding. Bipartite MDS is highly susceptible to large outliers, which can corrupt the largest singular vectors of the squared-distance matrix [Fig. 3(a)]. In contrast, SDP minimizes the sum of absolute (unsquared) deviation [38], and such loss is far more robust against gross corruptions. Metric MDS exhibits intermediate behavior, although we note that the choice of loss function heavily influences this behavior (Fig. 11 in Appendix A 8).

Lastly, we explore each algorithm’s tolerance to distances given as upper or lower bounds, which can arise when an experiment measures a value outside of its dynamic range. Figure 3(b) shows the embedding from the same distance matrix, now modified to represent 30% of measurements as upper or lower bounds. In this complete and noise-free case, both metric MDS and SDP can directly utilize these bounds to generate near-perfect reconstructions. In contrast, bipartite MDS cannot directly incorporate bounded data, and hence we replace each bounded measurement by the bound itself, which leads to worse reconstruction.

## IV. ANALYSIS OF ANTIBODY-VIRUS MEASUREMENTS

We next applied these embedding algorithms to an influenza dataset where the neutralization from 27 stem antibodies was measured against 49 viruses that circulated between 1933 and 2019 (Fig. 10 in Appendix A 8) [28]. The following section describes how to transform these experimental measurements into map distances and embed these antibody-virus interactions. Subsequent sections utilize this embedding to predict unmeasured interaction and quantify the degeneracy of the antibody response. We note that quantifying degeneracy would require thousands of experiments, yet such tasks become computationally tractable through these embeddings.

### A. Transforming antibody-virus measurements into distances

For each antibody-virus pair, the inhibitory concentration required to neutralize 50% of virus particles (IC_50_ in molar units) is measured, with lower values signifying a more potent antibody [39]. IC_50_s ranges from 8.6 × 10^−11^ *M* (very strong neutralization) to >1.6 × 10^−7^ *M* (weak neutralization outside the range of the assay).

To briefly describe the biological context for this dataset, each of the 27 antibodies targets the stem region of hemagglutinin, one of the key surface proteins on the influenza virus. This stem domain is highly conserved, and antibodies targeting it can neutralize very diverse viruses; for example, some antibodies measurably neutralize both the H1N1 and H3N2 influenza subtypes, which is rarely seen in antibodies targeting the head domain of this same viral protein [40].

Yet, even these broadly neutralizing antibodies have limits. Antibodies that potently neutralize H1N1 viruses tend to weakly neutralize H3N2 strains (and vice versa), while antibodies that neutralize all viruses tend to have intermediate effectiveness. These trends hint that there is an underlying trade-off between antibody potency (how much a virus is neutralized) and breadth (how many diverse viruses can be neutralized). Such patterns are difficult to directly discern from a table of pairwise interactions, yet they naturally emerge through an embedding.

**FIG. 4.**
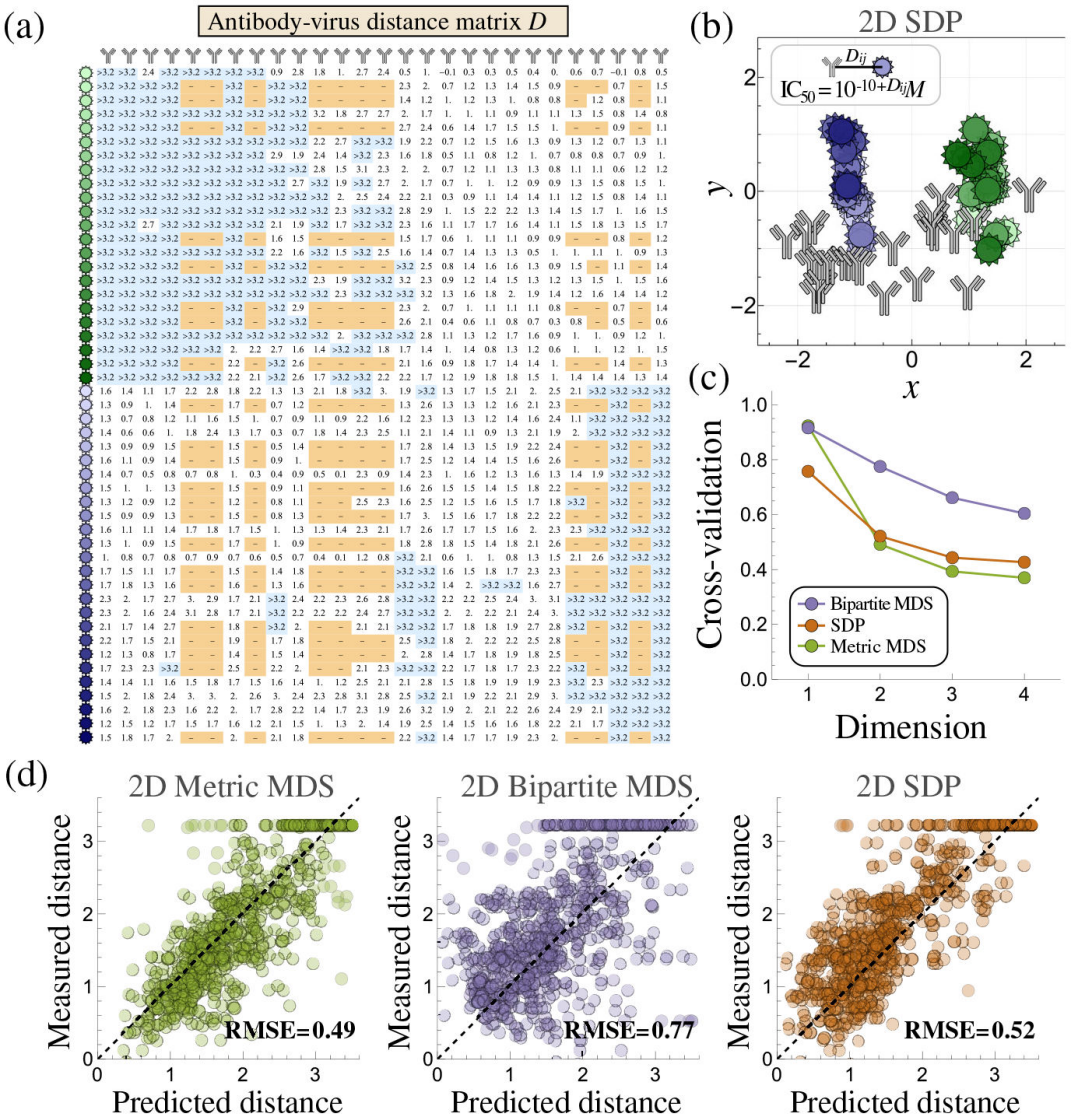
Mapping influenza antibody-virus interactions. (a) Experimentally measured distance matrix between 27 antibodies and 49 influenza viruses [39]. (b) The metric MDS embedding in 2D. (c) Tenfold cross-validation RMSE (calculated using the distance matrix). (d) Example of 2D cross-validation for each method, demonstrating that metric MDS performs the best.

Building off previous efforts [27,41], we first convert these antibody-virus neutralization measurements into distances. Previously, ordinal MDS demonstrated that antibody-virus interactions should be log transformed to obtain distances [42]. Antibodies typically have IC_50_s > 10^−10^*M* (since selection does not act below this point [43,44]), and hence we define antibody-virus distance as $D_{ij}=\log_{10}({\text{IC}}_{50}∕10^{-10}M)$ [Fig. 4(a)]. As described previously, a necessary condition for a Euclidean embedding is for the antibody-virus interactions to satisfy a modified triangle inequality (see Fig. S6 of Ref. [28]); indeed, we perform 400 000 tests of the triangle inequality on this dataset and find that it is satisfied in 99.7% of cases given the twofold error of the neutralization assay (with the remaining cases likely caused by rarer-but-larger experimental errors).

We then apply all three embedding algorithms to create a global map of the system. Since both the dimensionality and the ground truth coordinates are not known, we assess each algorithm through cross-validation by withholding 10% of the antibody-virus measurements, creating an embedding that predicts these withheld values, and then computing the RMSE of the difference between the predictions and measurements (repeating the process 10 times to minimize bias in the choice of withheld values). Five antibodies and five viruses whose positions could not be precisely fixed are removed from the dataset to ensure a rigid solution (Appendix A6).

None of the methods perform well in d=1 dimensions, while metric MDS slightly outperforms SDP in all higher dimensions. Both curves exhibit an “elbow” at d=2, suggesting that a 2D landscape captures the underlying structure of the system [Fig. 4(c)], as has been observed in other influenza datasets [27,41]. We note that the 2D cross-validation RMSE is ≈0.5 [Fig. 4(d)], so that withheld neutralization measurements are predicted within 10^0.5^≈ threefold, comparable to the noise of the neutralization assay.

### B. Designing optimal antibody cocktails

The resulting map provides a powerful way to computationally explore the efficacy of antibody combinations [Fig. 4(b)]. For example, the H1N1 viruses (green) and H3N2 viruses (blue) cluster together, as expected based on their genetic similarity. Interestingly, the centers of these clusters are ≈2.5 map units apart, demonstrating that while antibodies can be highly potent against H1N1 or H3N2 viruses, no antibody in the panel could strongly neutralize both subtypes.

Similar to the color wheel example in Fig. 1(a), the antibody-virus embedding not only represents the entities in this specific dataset, but also describes other potential antibodies and viruses (presuming they conform to the underlying structure of the embedding). For such entities, the embedding serves as a discovery space to quantify and constrain their behavior.

For example, within this framework we can design a mixture of n antibodies that optimally neutralizes the 5 viruses at the top of the H1N1 cluster as well as the 5 viruses at the top of the H3N2 cluster as potently as possible (Fig. 14 in Appendix A 8). This question lies at the heart of ongoing efforts to find new broadly neutralizing antibodies, yet few methods exist to predict or even constrain antibody behavior. To that end, we use each point on the map to describe a potential antibody whose neutralization against each mapped virus is determined by its map distance. This reduces the complex biological problem of enumerating antibody behavior to a straightforward geometry problem.

The theoretical best n=1 antibody mixture against these 10 viruses is represented by the center of the smallest circle that covers every virus [Fig. 14 in Appendix A 8, distance ≤ 1.4 (IC_50_ ≤ 10^−8.6^ *M*) for each virus]. For a mixture with n=2 antibodies, the potency can dramatically improve by using one H1N1-specific antibody and one H3N2-specific antibody [distance ≤ 0.3 (IC_50_ ≤ 10^−9.7^M) for each virus]. This problem can be readily extended to mixtures with an arbitrary n antibodies covering any set of mapped viruses. Given the growing number of efforts to find broadly neutralizing antibodies [45-48], it is essential to have some framework to estimate the limits of antibody behavior. Such estimations inform when the antibodies already discovered are near the theoretical best behavior (and, hence, further searching is less likely to lead to significant improvement) or when there are alleged antibodies that could perform orders of magnitude better than what we have currently seen [28].

### C. Degeneracy of the antibody response

Another key unexplored feature of the antibody response is its degeneracy: can the neutralization from a mixture of n antibodies behave like a mixture with fewer antibodies? For example, many vaccination regiments aim to elicit a broadly neutralizing antibody that will be potent against diverse viral strains. Yet, even if a postvaccination antibody response is measured against a large array of viruses, it may be impossible to determine whether its breadth is conferred by a single antibody or is due to the collective action of multiple antibodies. These questions hint at an underlying gap in our knowledge, namely, quantifying when antibody mixtures “unlock” fundamentally new behaviors that cannot be achieved by any individual antibody. Moreover, these topics are difficult to tackle experimentally, since the low-throughput neutralization assay is time and resource intensive.

Nevertheless, quantifying the degree of antibody degeneracy becomes tractable through an embedding. Such analyses necessarily make the strong assumption that every point on the map represents a viable antibody. Moreover, there may be other antibody phenotypes (e.g., from highly specific hemagglutinin head-targeting antibodies) that are not represented by any point on the map; in essence, the embedding serves to locally extrapolate antibody behavior based on the specific interactions provided as input [Fig. 4(a)]. Yet, with these caveats, we can explore how often a mixture made within this space of antibodies can be mimicked by a single antibody.

We describe an antibody mixture by n points in Fig. 4(b), with the ith antibody neutralizing the jth virus with an ${\text{IC}}_{50}^{ij}=10^{-10+D_{ij}}$ dictated by the map distance $D_{ij}$ between the antibody and virus. Since all antibodies in our panel bind to the same region of the hemagglutinin stem [49-51], we treat their binding as competitive, so only one antibody can bind to each hemagglutinin monomer at a time. Thus, a mixture’s neutralization against virus j is given by

(11) $${\text{IC}}_{50}^{\text{mixture}}={\left(\sum_{i}\frac{f_{i}}{{\text{IC}}_{50}^{ij}}\right)}^{-1},$$

where $f_{i}$ represents the fraction of antibody i in the mixture (with $\sum_{i}f_{i}=1$). A diluted antibody with small $f_{i}$ will effectively have a weaker (larger) IC_50_, which in the embedding translates to an extra “distance handicap” of $f_{i}$ added to its distance from any virus. We note that this binding model has been verified on antibody mixtures from this specific panel [28] and on other datasets [52,53]. For simplicity, we restrict ourselves to equimolar n-antibody mixtures ($f_{i}=1∕n$).

**FIG. 5.**
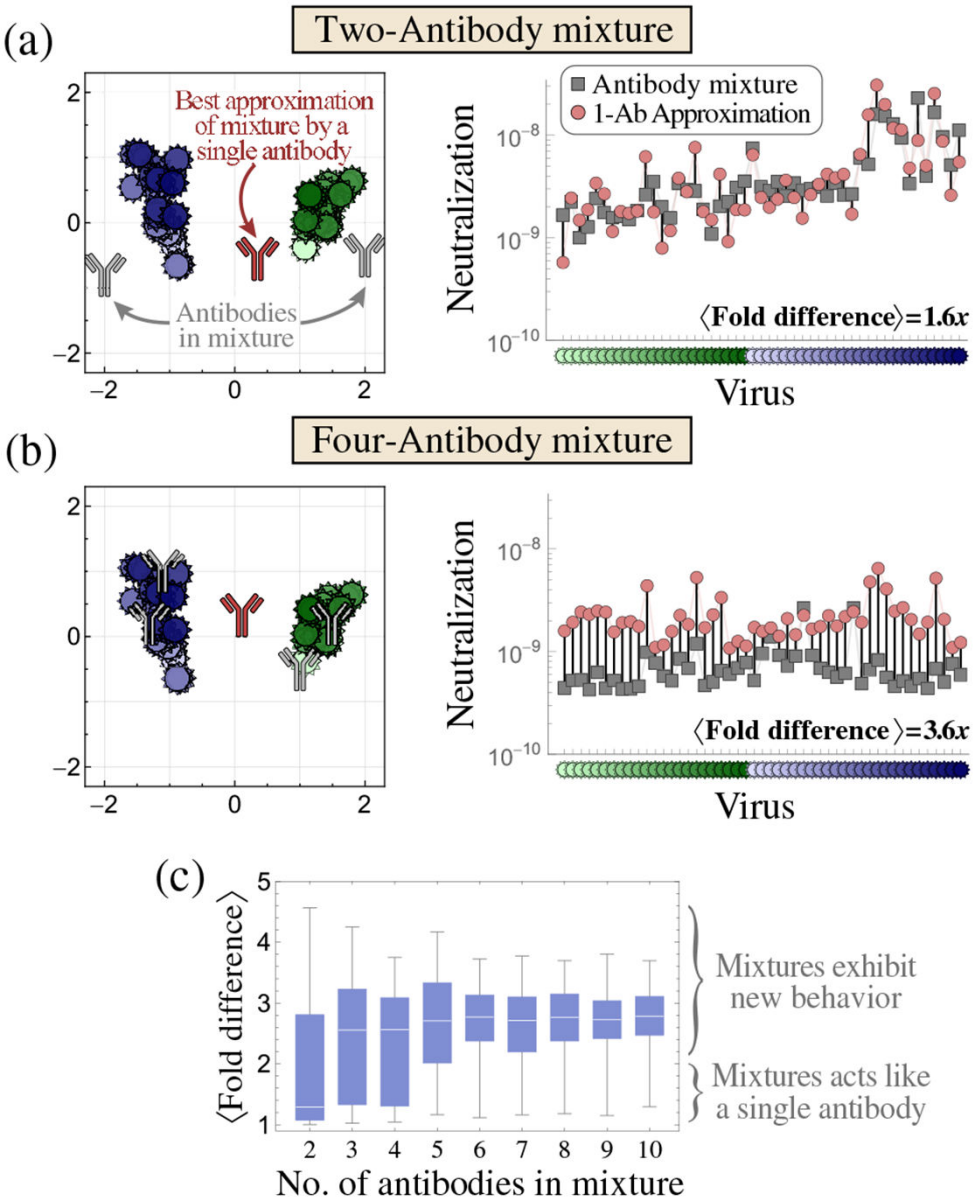
Degeneracy of antibody mixtures. Examples of (a) a two-antibody mixture that behaves like a single antibody [1-Ab approximation] and (b) a four-antibody mixture that exhibits distinct behavior from any individual antibody. Left: the antibodies in the mixture (gray) and the best approximating antibody (red). Right: the neutralization IC_50_s across all viruses. The fold difference between the mixture and antibody is shown by the vertical black lines for each virus, with the mean fold difference given in the bottom right. (c) For each mixture containing n antibodies (x axis), we sample 100 equimolar mixtures and quantify their average fold difference to the nearest approximating antibody.

Given a specific mixture (n random points on the map, sampled near the H1N1 and H3N2 clusters), we quantify the closest approximating single antibody (another point on the map) by scanning through every possible location and minimizing the average fold difference between the mixture’s and antibody’s neutralization profiles across all viruses. Figure 5(a) shows a mixture of two antibodies (gray), one of which is potent against the blue H3N2 viruses on the left of the map and the other potent against the green H1N1 viruses, that behave nearly identically to a single antibody (red) in the middle of the map. While a few viruses are neutralized differently by the mixture and antibody [vertical black lines, right-hand panel of Fig. 5(a)], on average the antibody’s IC_50_s are within 1.6-fold of the mixture’s values against these 50 diverse viruses. This discrepancy is comparable to the ≈twofold error of the assay, and, hence, given either neutralization profile, we cannot determine whether it arises from an individual antibody or a mixture.

Higher-order mixtures unlock more unique behaviors that cannot be replicated by an individual antibody. For example, not only does the four-antibody mixture in Fig. 5(b) show a 3.6-fold difference from the nearest approximating antibody, but the mixture’s measurements are systematically lower across nearly all viruses. Thus, neutralization profiles exhibiting such strong breath are indicative of multiple antibodies.

To systematically explore degeneracy, we sample 100 antibody mixtures for each n (with 2≤n≤10) and find the closest approximating single antibody. The resulting distributions of the mean fold difference are shown in Fig. 5(c). While two-antibody mixtures tend to resemble individual antibodies, higher-order mixtures often exhibit distinctive profiles with a ⟨fold difference⟩ > 2 to the closest approximating antibody. By the time n≥5 antibodies are combined, the likelihood that they match any single antibody becomes exceedingly rare.

## V. DISCUSSION

Embedding algorithms fill a “hole” in our understanding by transforming local pairwise interactions into a global map. Such algorithms have been used to identify when a new viral variant arises, quantify drug-protein interactions, and distinguish between cell types [27,54,55]. Yet we propose that such algorithms also provide the groundwork for new theoretical studies such as quantifying antibody degeneracy that only become possible when we reveal the underlying structure of a system.

In the context of antibody-virus interactions, an embedding provides a rigorous approach to extrapolate available measurements. Each point describes a potential antibody, and the entire map defines a basis set of antibody behaviors. By coupling these data-driven results with a biophysical model of how antibodies collectively act, we can model higher-order mixtures and pave the way to study the complex array of antibodies within each person. For example, our degeneracy analysis compares a four-antibody cocktail [one of $(\begin{array}{c} 27\\ 4 \end{array})\approx 18\;000$ possible mixtures given our antibody panel] against all predicted singleantibody behaviors. In doing so, we leverage the combinatorics of antibody combinations to explore the vast space of antibody mixtures.

Such analysis implicitly assumes that a dataset can be rescalable into a lower dimension. This claim can be verified through cross-validation, by using multiple complementary approaches (e.g., embeddings, low-rank matrix completion) to detect a simple underlying structure, or by computing the rank of a matrix-complete dataset (for this antibody dataset the top 3 singular values account for 90% of the variance). We further note that multiple datasets measuring the serum response (i.e., the array of antibodies within an individual’s blood) found low-dimensional signatures [27,41,56,57], and hence we expect the response of individual antibodies should be similarly low dimensional.

While metric MDS has been used to embed the interactions between influenza viruses and serum [27,41], the dataset we analyze in this paper contains individual antibodies that all target the same site on the virus, namely, the hemagglutinin stem. This distinction is important, since embedding antibodies targeting multiple sites leads to poorer cross-validation RMSE (Fig. 15), suggesting that the structure of the neutralization landscape can differ for each viral epitope, potentially necessitating a different embedding for each site.

Although embedding via numerical minimization (metric MDS) is flexible and straightforward to implement, alternate methods that leverage the desired structure of the data (bipartite MDS and SDP) may perform better in certain regimes. Moreover, such techniques may scale better for larger datasets, and hence can be used instead of (or in combination with) metric MDS to yield fast, robust embeddings. For any dataset, these methods can be compared head to head through cross-validation on a subset of data.

**FIG. 8.**
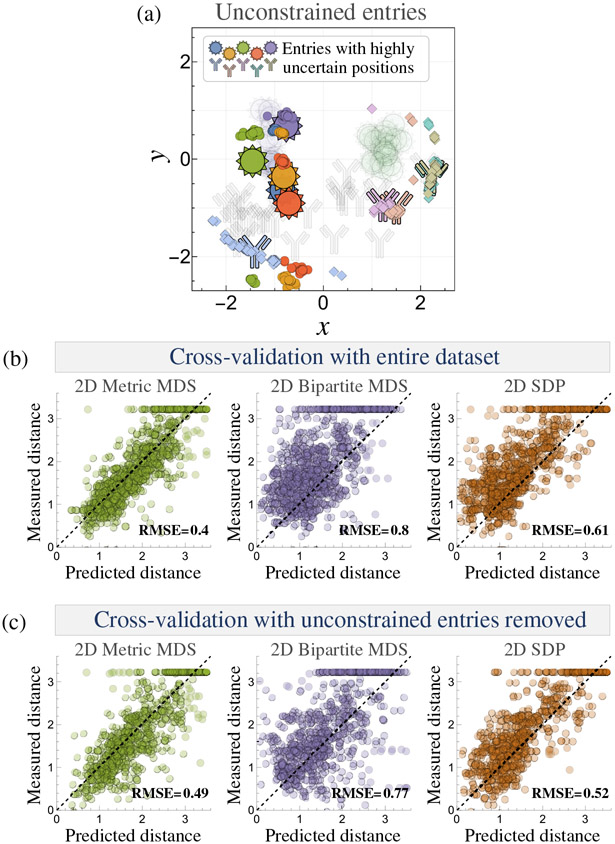
Creating a rigid embedding by removing unconstrained entries. (a) We create 76 embeddings (withholding each possible antibody or virus) and analyze how tightly clustered each entry is across all embeddings. The five antibodies (02-1B02, 22-1B08, 55-1D06, 58-6F03, CR6261) and five viruses (H3N2 A/Moscow/10/1999, A/Indiana/10/2011, A/Switzerland/9715293/2013, A/Hong Kong/4801/2014, A/Singapore/INFIMH-160019/2016) with the largest scatter are highlighted, with the average position of all remaining entries shown with small opacity. (b) Cross-validation [as shown in Fig. 4(d)] using 2D embeddings on the full dataset. (c) Cross-validation using 2D embeddings on the partial dataset with the 10 entries from (a) removed.

**FIG. 9.**
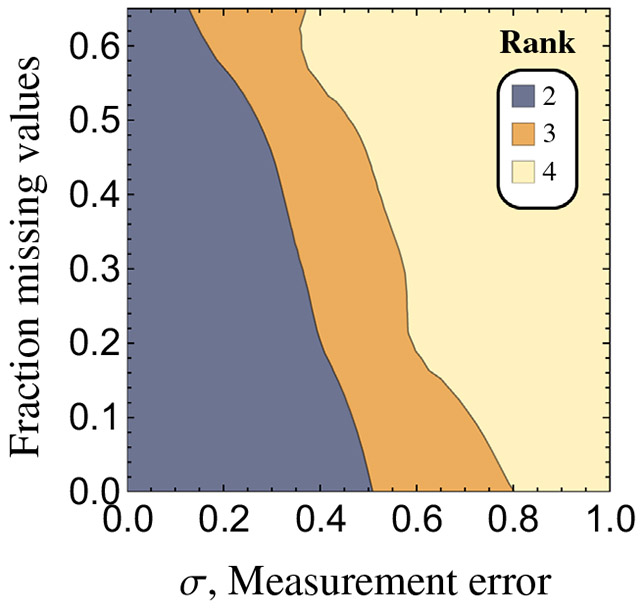
Creating a rigid embedding by removing unconstrained entries. (a) We make 76 embeddings (withholding each possible antibody or virus) and analyze how tightly clustered each entry is across all embeddings. The five antibodies and five viruses with the largest scatter are highlighted, with the average position of all remaining entries shown with small opacity. (b) Cross-validation [as shown in Fig. 4(d)] using 2D embeddings on the full dataset. (c) Cross-validation using 2D embeddings on the partial dataset with the 10 entries from (a) removed.

**FIG. 10.**
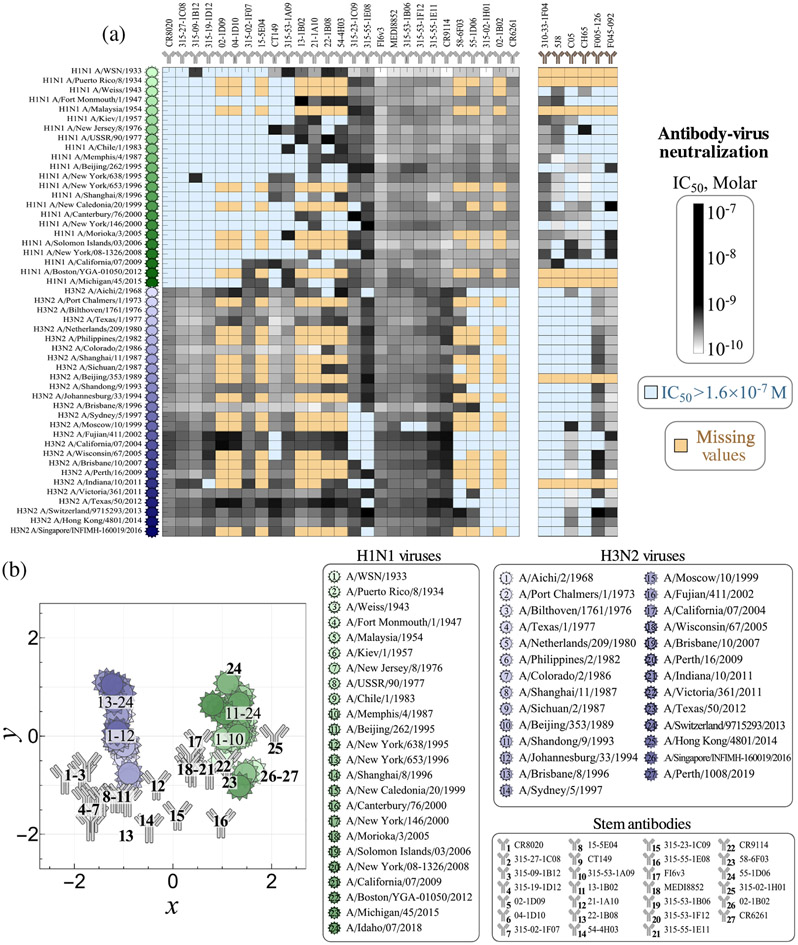
Annotated influenza antibody-virus data from Creanga *et al.* [39]. (a) Neutralization measurements of 49 influenza viruses against 27 antibodies targeting the hemagglutinin stem (gray) and 6 antibodies targeting hemagglutinin head (brown). The inhibitory concentration of antibody needed to neutralize 50% of viruses (IC_50_, gray scale). Some antibody-virus interactions are not measured (tan), and some antibodies exhibit weak neutralization (IC_50_ > 1.6 × 10^−7^ *M*, light blue) outside the dynamic range of the assay. (b) The same 2D metric MDS embedding [as in Fig. 4(b)] with the antibodies and viruses labeled.

**FIG. 11.**
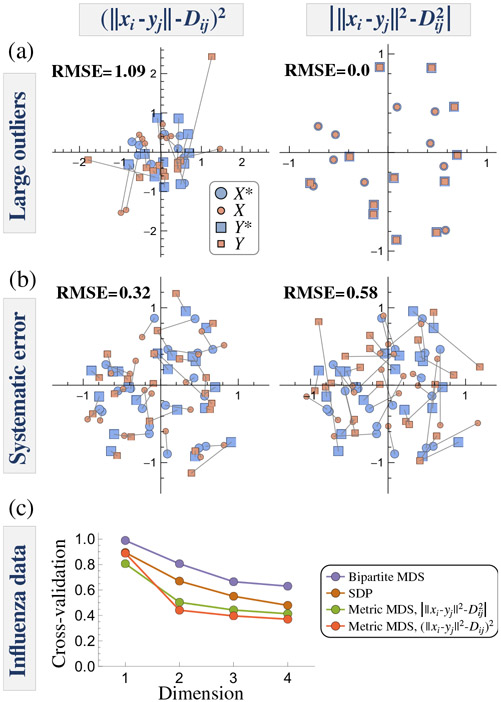
Choice of loss function strongly influences metric MDS. We compare two loss functions for metric MDS. Left: mean squared error between unsquared distances (shown in the main text). Right: mean absolute error between the squared distances. (a) Mean absolute error handles the distance matrix with large outliers far better [see Fig. 1(a)]. (b) Large systematic noise is handled better by mean squared error, since this represents the maximum likelihood estimator for approximately Gaussian error (see σ=1, $f_{\text{missing}}=0$ from Fig. 2). (c) Cross-validation for the influenza data in Fig. 4 is slightly lower for metric MDS with mean squared error for embeddings with dimension ≥ 2.

**FIG. 12.**
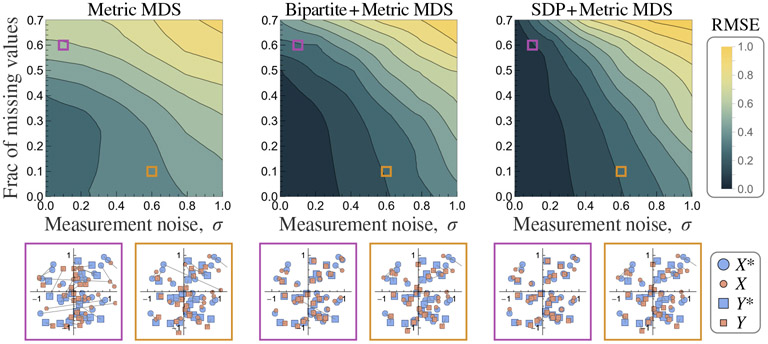
Postprocessing an embedding with metric MDS. As in Fig. 2, data are simulated with elementwise noise σ and a fraction $f_{\text{missing}}$ of missing entries. The results of each embedding are used to initialize one additional metric MDS, which greatly improves its accuracy. Error is computed as the average Euclidean distance between the numerical and actual coordinates (aligned using a rigid transform). Example plots at the bottom show an embedding when σ=0.1 and $f_{\text{missing}}=0.6$ (purple box) as well as σ=0.6 and $f_{\text{missing}}=0.1$ (brown box) for each method.

**FIG. 13.**
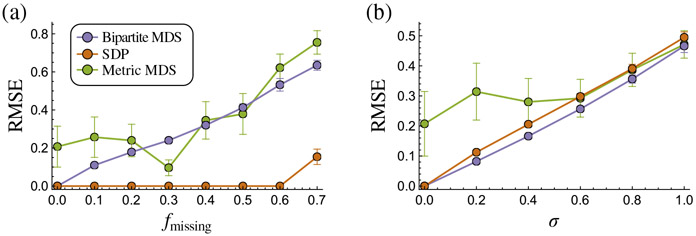
Embedding for noise-free or complete datasets. (a) The noise-free limit σ=0 and (b) the limit of complete data $f_{\text{missing}}=0$. In both panels, m=n=20 and the error represents the RMSE of Euclidean distances between the estimated and true coordinates (once aligned via a rigid transform).

**FIG. 14.**
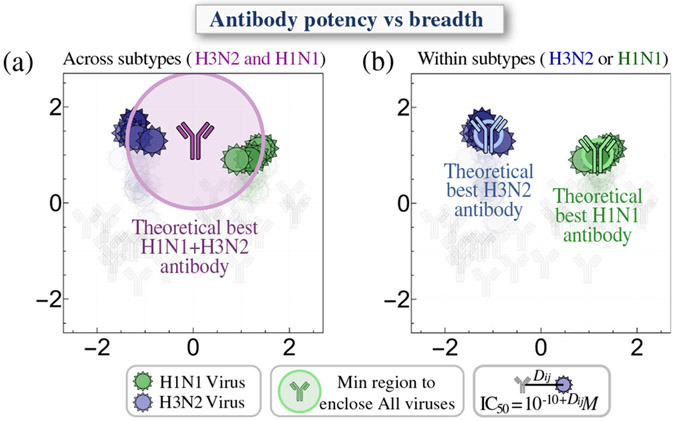
Predicting optimal neutralization for any mapped viruses. Suppose we want to neutralize the five viruses at the tops of the H1N1 and H3N2 clusters in Fig. 4(b) (H1N1 A/New York/638/1995, A/Beijing/262/1995, H1N1 A/New Caledonia/20/1999, A/Canterbury/76/2000, A/New York/146/2000 and H3N2 A/Fujian/411/2002, A/California/07/2004, A/Indiana/10/2011, A/Texas/50/2012, A/Perth/1008/2019). Using the points on the map to represent potential antibody neutralization profiles, we determine (a) the best single antibody or (b) the best two-antibody mixture that would neutralize these viruses the most potently (with the smallest possible distance between any virus and the nearest antibody). The solution is given by the n minimum covering circles for these viruses, with the antibodies positioned at the centers of each circle. Figure is adapted from Ref. [28].

**FIG. 15.**
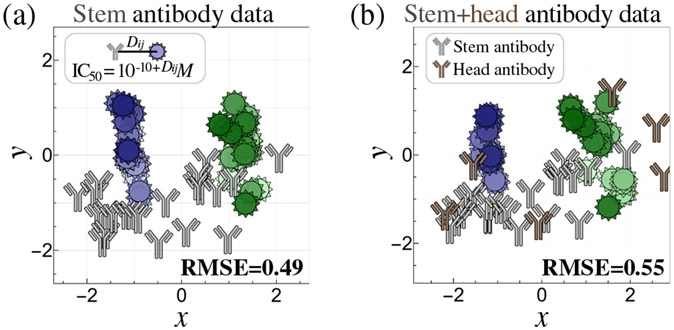
Embedding antibodies targeting one versus multiple viral epitopes. (a) Embedding the 27 antibodies [gray, shown in Figs. 4(a) and 4(b)] targeting a single site on the influenza virus, namely, the hemagglutinin stem. (b) In addition to these stem antibodies, we embed 6 additional antibodies (brown) targeting different sites on the hemagglutinin head. The cross-validation RMSE (shown in the bottom right) is larger when including these head antibodies. Neutralization data for all antibodies are given in Fig. 1(a).

More work is needed to understand the limits of these embeddings and quantify their predictive power. A key aspect of such embeddings is their rigidity, which determines whether entries can be precisely fixed by the available data (Fig. 8) [14]. We hypothesize that other universal rigidity criteria may exist for certain bipartite graphs depending on their connectivity, where low-rank matrix completion techniques can be leveraged to provide guarantees for exact recovery in SDP approaches.

We are just beginning to scratch the surface on aspects of the antibody response that can be probed with these embeddings, from designing antibody cocktails to determining how the antibody response evolves on the map with each viral exposure. As datasets continue to grow in size and complexity, it becomes increasingly important to quantitatively visualize interactions between entities. Future datasets may require multilocalization, where higher-order interactions (e.g., between a ligand and multimeric receptor [24]; antibodies, antigens, and cell receptors [58]; or single-cell multiomics datasets [59]) are embedded in a low-dimensional space.

For reproducibility, example codes for each algorithm and the complete *Mathematica* code are available [60].

## Figures and Tables

**FIG. 6.**
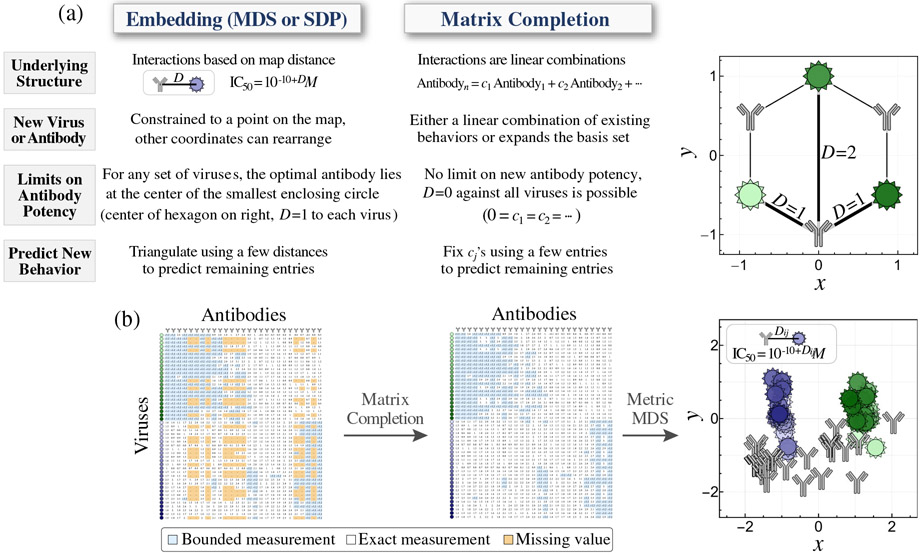
Differences between Euclidean embedding algorithms and low-rank matrix completion. (a) Embedding and matrix completion algorithms assume different underlying structures, with the former constraining points to lie in Euclidean space where antibody-virus distance (D) dictates each interaction, whereas the latter searches for the smallest basis set of behaviors whose linear combinations describe all antibodies and viruses. With an embedding, a new entry is described by a point on the map; other entries may change positions, although there is minimal rearrangement once there are many mapped entries. In contrast, matrix completion attempts to characterize new antibodies as linear combinations of existing antibodies, but when this is not possible the basis set is expanded. As a result, an embedding imposes strong limits on new antibody behavior (e.g., for the configuration shown, an antibody with the shortest possible distance to all three viruses lies at the center of the hexagon, with D=1 to all three viruses), whereas matrix completion allows an antibody to adopt the (unrealistic) case of extreme potency D=0 against every virus. Both methods are able to use a few measurements from a new antibody or virus to predict the interactions with all other mapped entries. (b) For datasets where matrix completion outperforms a direct embedding, we first matrix-complete any missing values and then embed the resulting complete data. This procedure is shown for the antibody-virus dataset in Figs. 4(a) and 4(b).

**FIG. 7.**
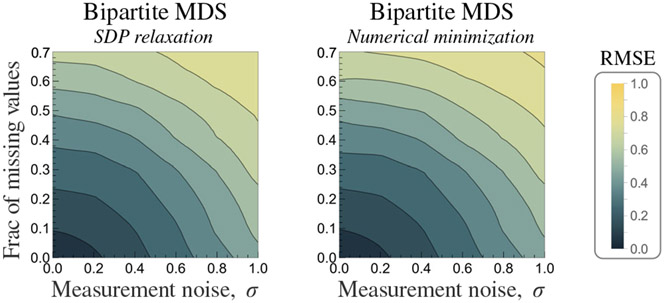
Comparing methods to determine $A_{U}$ and $A_{V}$ in bipartite MDS. Left: reproducing Fig. 2 from the main text (middle panel), where in each simulation $A_{U}$ and $A_{V}$ are computed using the SDP approach via Eq. (A7). Right: numerically minimizing using Eq. (2) yields nearly identical results.

## APPENDIX:

In this appendix, we compare embedding and matrix completion methods as well as describe the implementations of metric MDS, bipartite MDS, and SDP algorithms.

### 1. Solving the classical localization problem for complete, noise-free data

Here, we present the well-known solution to the (monopartite) classical localization problem, where the noise-free distances $D\in R^{n\times n}$ is provided between every pair of n points in d dimensions. Our goal is to determine the coordinates ${\left\{x_{i}\right\}}_{i=1}^{n}\subset R^{d}$ such that $D_{ij}=\|x_{i}-x_{j}\|$.

Since an embedding always has a translational degree of freedom, we assume without loss of generality that the coordinates are centered around the origin, $\sum_{i=1}^{n}x_{i}=0$. We define the combined coordinate matrix $X={\left[x_{1},\ldots ,x_{n}\right]}^{T}\in R^{n\times d}$. Note that the entrywise squared-distance matrix can be written as

(A1)
$$D\;\circ \;D=\text{diag}(XX^{T})1_{n}^{T}+1_{n}\text{diag}{\left(XX^{T}\right)}^{T}-2XX^{T},$$

where the first two terms on the right-hand side are outer products.

The algorithm proceeds in two steps. First, we apply the centering matrix $J_{n}$ [Eq. (3)] from the left and right to row center and column center the squared distances,

(A2)
$$-\frac{1}{2}J_{n}(D\;\circ \;D)J_{n}=XX^{T},$$

where we use the fact that $J_{n}1_{n}=0$ and $1_{n}^{T}J_{n}=0$. This transforms the distance matrix into a matrix of inner products for the coordinates $XX^{T}$.

The second step is to compute the SVD of the left-hand side, ${U\Sigma V}^{T}=XX^{T}$, which will only have d nonzero singular values. From this form, we can immediately read out the solution $X={U\Sigma }^{1∕2}$.

### 2. Comparing embedding algorithms with matrix completion

In this section, we compare the embedding algorithms used in this work with low-rank matrix completion algorithms. One key difference between these approaches is that an embedding projects entries into d dimensions where map distance is related to each experimental measurement, whereas matrix completion searches for the smallest basis set of behaviors whose linear combinations describe the data. As a result, embedding techniques impose a limit on how good an antibody can be against multiple viruses, whereas matrix completion approaches always allow an antibody to be maximally potent against all viruses [Fig. 6(a)].

In addition, when embedding into d=2 dimensional space, our SDP algorithm utilizes a rank-d Gram matrix G, whereas matrix completion on antibody-virus data (analogous to a distance matrix) would be at best rank d+2 with perfect, noise-free data (see Table S1 of Ref. [61], ranks ranged from 6 to 23). Thus, embeddings utilize a simpler structure that requires fewer parameters. Moreover, the computationally cheaper bipartite MDS (a spectral method based on SVD) is in line with the latest trends in nonconvex matrix completion [62] which are at best rank d.

However, it is hard to know *a priori* which method will be superior for a given dataset, yet it is easy to assess all methods through cross-validation. In cases where matrix completion performs better than direct embedding, a dataset can first be matrix completed and then embedded. Indeed, we found that inferring the missing antibody-virus interactions using matrix completion (cross-validation RMSE = 0.34) outperforms any of the embedding techniques in 2D, yield comparable results to 4D metric MDS [cross-validation RMSE = 0.37, Fig. 4(c)]. This suggests that matrix completion should first precomplete this dataset before an embedding.

Hence, these algorithms should not just be explored individually, but in combination. Initialization with matrix completion offsets a key shortcoming of bipartite MDS, namely, its inability to handle missing values. Figure 6(b) demonstrates the resulting matrix completion followed by metric MDS for the antibody-virus dataset we analyze in this work, using a low-rank matrix completion algorithm from Ref. [61]. For simplicity, we handle bounded values by first replacing them by their bounded measurement, matrix completing, transforming into map distance (10 + log[IC_50_/1*M*]), and finally replacing all values greater than 3.2 into bounded measurements. More sophisticated algorithms can directly incorporate this bounded information into the matrix completion step [63].

### 3. Handling missing values in the distance matrix

In metric MDS and SDP, missing values are automatically ignored, since the sums in (2) and (10) are over the measured distances (i,j)∈ℰ. In other words, each measured edge constrains the embedding while unmeasured edges are ignored. Bipartite MDS requires a complete matrix to compute the SVD, and hence each missing entry is first filled in using the mean of all nonmissing measurements in its row and column.

### 4. Handling upper or lower bounds in the distance matrix

Sometimes measurements are given as upper or lower bounds on $\left\|x_{i}-y_{j}\right\|$ (signifying weak or strong interactions outside the dynamic range of the experiment).

For an upper bound $\|x_{i}-y_{j}\|<b_{\text{up}}$ in metric MDS, we modify the relevant summand in the loss function to $(1∕1+e^{c(b_{\text{high}}-\|x_{i}-y_{j}\|)}){\left(b_{\text{high}}-\|x_{i}-y_{j}\|\right)}^{2}$, where c>10 is a positive constant (in this work, we choose c=10 based on the scale of the distance measurements). The prefactor in the summand penalizes violations of the bound while minimally increasing the loss when the bound is satisfied. For a lower bound, $\|x_{i}-y_{j}\|>b_{\text{low}}$, we similarly modify the summand to $(1∕1+e^{-c(b_{\text{low}}-\|x_{i}-y_{j}\|)}){\left(b_{\text{low}}-\|x_{i}-y_{j}\|\right)}^{2}$. Note that the resulting cost function is nonconvex, which can prevent numerical algorithms from finding a good minimizer. When computing RMSE in the cross-validation analysis [Figs. 4(c) and 4(d)], we add these same prefactors when the measured distance is an upper or lower bound.

Bipartite MDS requires exact distance measurements to compute a SVD, and hence we replace each bounded measurement by the bound itself, which leads to poorer embeddings.

In SDP, bounded values are handled by modifying the second constraint in Eq. (10). For example, an upper bound $\|x_{i}-y_{j}\|<b_{\text{up}}$ is enforced by the one-sided constraint ${\left(G_{11}\right)}_{ii}-2{\left(G_{12}\right)}_{ij}+{\left(G_{22}\right)}_{jj}-b_{\text{up}}^{2}<E_{ij}$. A lower bound $\|x_{i}-y_{j}\|>b_{\text{low}}$ is enforced by the one-sided constraint $-E_{ij}<{\left(G_{11}\right)}_{ii}-2{G_{12})}_{ij}+{\left(G_{22}\right)}_{jj}-b_{\text{low}}^{2}$. The objective remains the same, namely, to minimize the sum of (positive) errors $E_{ij}$ between the embedding and distance measurements.

### 5. Determining the affine transformation in bipartite MDS

In this section, we provide some intuition for bipartite MDS and describe in detail the final SDP step that determines the affine transform between X and Y.

We begin by rewriting Eq. (4) as

(A3)
$$D^{*}\;\circ \;D^{*}=\text{diag}(X^{*}X^{*T})1_{n}^{T}+1_{m}\text{diag}{\left(Y^{*}Y^{*T}\right)}^{T}-2X^{*T}Y^{*}.$$

Because $1_{m}$, $1_{n}$ lie in the null space of $J_{m}$, $J_{n}$, double centering isolates the inner product term as in Eq. (5). Using the rank-d SVD ${U\Sigma V}^{T}=-\frac{1}{2}J_{m}(D^{*}\;\circ \;D^{*})J_{n}$, we can rewrite Eq. (5) as

(A4)
$$X^{*}=U\Sigma (V^{T}{\left(Y^{*T}J_{n}\right)}^{†}),\;J_{n}Y^{*}=V(\Sigma U^{T}{\left(X^{*T}\right)}^{†}).$$

where ‘‘†’’ denotes the pseudo-inverse. This reveals that the embedding $X^{*}$, $Y^{*}$ can be determined up to linear transforms as in Eqs. (6) and (7).

As we describe in the main text, the final step of bipartite MDS is to determine the affine transforms $A_{U}$, $A_{V}$ (satisfying $A_{U}A_{V}^{T}=I_{d}$) and the translation $t_{V}$, so that the embeddings $X^{*}$, $Y^{*}$ in Eqs. (6) and (7) match the distance matrix. This can be done numerically either by minimizing the loss function in Eq. (2) that optimally handles systematic noise or by minimizing the loss function of squared distances,

(A5)
$$\underset{{\left\{x_{i}\right\}}_{i=1}^{m},{\left\{y_{j}\right\}}_{j=1}^{n}}{\text{min}}\sum_{(i,j)\in ℰ}|D_{ij}^{2}-{\left\|x_{i}-y_{j}\right\|}^{2}|,$$

that better handles outliers (Fig. 11).

Instead of numeric minimization, we can use semi-definite programming to solve Eq. (A5) and prevent the minimization from getting stuck at local minimum. To that end, we construct the (2d+1)×(2d+1) Gram matrix,

(A6)
$$\tilde{G}=\left(\begin{array}{ccc} - & A_{U} & -\\ - & A_{V} & -\\ - & t_{V}^{T} & - \end{array}\right)\left(\begin{array}{ccc} | & | & |\\ A_{U}^{T} & A_{V}^{T} & t_{V}\\ | & | & | \end{array}\right),$$

with which we can express ${\left\|x_{i}-y_{j}\right\|}^{2}$. Using Eqs. (6) and (7), we can write

(A7)
$${\left\|x_{i}-y_{j}\right\|}^{2}=U_{i}\Sigma A_{U}A_{U}^{T}\Sigma U_{i}^{T}+V_{j}A_{V}A_{V}^{T}V_{j}^{T}+t_{V}^{T}t_{V}\;\;-2V_{j}\Sigma U_{i}-2U_{i}\Sigma A_{U}t_{V}+2V_{j}A_{V}t_{V}$$

in terms of the entries of $\tilde{G}$.

Define the minimization matrix $\gamma \in R^{m\times n}$ with $\gamma_{ij}=D_{ij}^{2}-{\left\|x_{i}-y_{j}\right\|}^{2}$. To minimize the absolute value of the $\gamma_{ij}$, we use Schur’s complement condition, defining the auxiliary matrix $\tilde{\gamma }\in R^{m\times n}$ and using semidefinite programming to solve

(A8)
$$\underset{\tilde{G},\tilde{\gamma }}{\text{minimize}}\;\;\sum_{(i,j)\in ℰ}{\tilde{\gamma }}_{ij}\;\text{subject to}\;\;\tilde{G}\;\underline{≻}\;0,\;\;\left(\begin{array}{cc} {\tilde{\gamma }}_{ij} & \gamma_{ij}\\ \gamma_{ij} & 1 \end{array}\right)\underline{≻}\;0,\;\;\forall \;1\leq i\leq m,1\leq j\leq n,\;\;{\tilde{G}}_{d+1:2d,1:d}=I_{d}.$$

We then use Cholesky decomposition to extract $A_{U}$ from ${\tilde{G}}_{1:d,1:d}$ and determine $A_{V}={\tilde{G}}_{d+1:2d,d+1:2d}A_{U}$ as well as $t_{V}={\tilde{G}}_{2d+1,d+1:2d}A_{U}$. The resulting embedding is given by Eqs. (6) and (7).

Because of the rank relaxation in this SDP approach, we note that the optimal affine transformations $A_{U}$ and $A_{V}$ are determined in a higher-dimensional space. While the global numeric minimizer of Eq. (A5) is in the SDP search space, the global SDP minimizer may be different. In practice, we find little difference between the two approaches (Fig. 7).

### 6. Assessing the rigidity of an embedding

To determine whether the antibody-virus embedding is rigid (i.e., if the embedding is unique or if multiple incongruent embeddings can describe this dataset), we remake the embedding 76 times (once with each of the 27 antibodies or 49 viruses removed) using 2D metric MDS. These embeddings are aligned through rigid transforms, and the position of each entry is compared across all embeddings.

While most entries are tightly constrained on the map [with an average standard deviation of 0.14 map units in either the x or y directions, comparable to experimental error of $\log_{10}(2)\approx 0.3$], we find 5 viruses and 5 antibodies whose position jumps by > 1 map unit in some embeddings [Fig. 8(a); removed entries named in caption], and these entries are removed before carrying out the embeddings in Fig. 4.

We note that when using the full dataset, metric MDS outperforms the bipartite MDS as well as SDP algorithms [Fig. 8(b)], whereas removing these uncertain entries leads to more rigid embeddings where metric MDS and SDP have comparable cross-validation [with both outperforming bipartite MDS, Fig. 8(c)]. This is consistent with the tendency of SDP approaches to fail when there are multiple possible solutions, suggesting that such rigidity analysis is paramount for these structured approaches. We hypothesize that the uniformly poor performance of bipartite MDS arises from the many missing and bounded values in the antibody-virus dataset, and hence that approach should be reserved for nearly complete datasets with little to no bounded measurements.

### 7. Numeric minimization in metric multidimensional scaling

Multiple methods exist to solve the distance geometry problem. One of the earliest methods uses stress majorization which is based on gradient descent [64]. Homotopy methods have also been proposed to tame the inherent nonconvexity [65]. We also note that the distance geometry problem arises in protein structural determination, where simulated annealing approaches such as XPLOR-NIH are commonly used [66].

For large graphs, local to global build-up approaches have been developed [67-69] along with convex relaxation approaches [70-72]. These methods often come with theoretical guarantees that assume a statistical model for the distance measurements. More recently, branch-and-bound-type algorithms have also been applied to specialized instances of the distance geometry algorithm [73].

In our recent work, we observed that when some distance measurements are grossly corrupted, global optimization techniques such as simulated annealing or basin hopping failed with multistart, although a more sophisticated convex relaxation approach succeeded in almost all situations [74]. This robust behavior encourages us to examine the use of convex optimization methods more broadly.

### 8. Notes on embedding antibody-virus data

Figure 10 shows the full antibody-virus data. Through embedding, we predict the optimal neutralization profile of a single antibody [Fig. 14(a)] or a two antibody mixture [Fig. 14(b)], where smaller covering circles represent exponentially stronger neutralization against the encompassed variants.

As noted in the main text, the choice of loss function can affect the resulting embedding (Fig. 11). While SDP exhibits the most robust behavior in the presence of noise and missing values (Fig. 13), postprocessing with metric MDS can yield better performance (Fig. 12). Note that with SDP, we can approximate the rigidity of the system through the rank of the positive-semidefinite matrix G (Fig. 9).
